# FTY720 Induces Autophagy-Associated Apoptosis in Human Oral Squamous Carcinoma Cells, in Part, through a Reactive Oxygen Species/Mcl-1-Dependent Mechanism

**DOI:** 10.1038/s41598-017-06047-9

**Published:** 2017-07-17

**Authors:** Li-Yuan Bai, Chang-Fang Chiu, Shih-Jiuan Chiu, Po-Chen Chu, Jing-Ru Weng

**Affiliations:** 1 College of Medicine, China Medical University, Taichung, 40402 Taiwan; 2 Division of Hematology and Oncology, Department of Internal Medicine, China Medical University Hospital, Taichung, 40447 Taiwan; 3 Cancer Center, China Medical University Hospital, Taichung, 40447 Taiwan; 4 School of Pharmacy, Taipei Medical University, Taipei, 11042 Taiwan; 5 Institute of Biological Chemistry, Academia Sinica, Taipei, 11529 Taiwan; 6 Department of Marine Biotechnology and Resources, National Sun-Yat-sen University, Kaohsiung, 80424 Taiwan

**Keywords:** Translational research, Pharmacology

## Abstract

In this study, we interrogated the mechanism by which the immunosuppressant FTY720 mediates anticancer effects in oral squamous cell carcinoma (OSCC) cells. FTY720 differentially suppressed the viability of the OSCC cell lines SCC4, SCC25, and SCC2095 with IC_50_ values of 6.1, 6.3, and 4.5 μM, respectively. This antiproliferative effect was attributable to the ability of FTY720 to induce caspase-dependent apoptosis. Mechanistic evidence suggests that FTY720-induced apoptosis was associated with its ability to inhibit Akt-NF-κB signaling, to facilitate the proteasomal degradation of the antiapoptotic protein Mcl-1, and to increase reactive oxygen species (ROS) generation. Both overexpression of Mcl-1 and inhibition of ROS partially protected cells from FTY720-induced caspase-9 activation, PARP cleavage and cytotoxicity. In addition, FTY720 induced autophagy in OSCC cells, as manifested by LC3B-II conversion, decreased p62 expression, and accumulation of autophagosomes. Inhibition of autophagy by bafilomycin A1 protected cells from FTY720-induced apoptosis. Together, these findings suggest an intricate interplay between autophagy and apoptosis in mediating the tumor-suppressive effect in OSCC cells, which underlies the translational potential of FTY720 in fostering new therapeutic strategies for OSCC.

## Introduction

Oral squamous cell carcinoma (OSCC) is the most common malignant tumor of the head and neck, and the incidence is increasing worldwide^1^. OSCC is associated with multiple risk factors, among which tobacco use, alcohol, and betel quid chewing are most noteworthy. In response, surgery, chemotherapy, radiotherapy, and molecular targeted therapy have been used for the treatment of OSCC. However, drug resistance, either intrinsic or acquired, poses a major obstacle for OSCC therapy in light of limited treatment modalities, which highlights the urgency to develop novel strategies for OSCC treatment.

FTY720 is a synthetic analogue of ISP-1 (myriocon), a fungal metabolite found in traditional Chinese herbal medicine^2^. Originally, FTY720 was developed as an immunosuppressant because of its activity, after being converted to FTY720-phosphate, to promote the migration and homing of lymphocytes through interaction with the sphingosine-1-phosphate (S1P) receptors^3–5^. Recently, the antitumor activity of FTY720 has been the focus of many investigations. FTY720 has also been shown to induce apoptosis in many different human cancer cell lines, including those of breast, prostate, lung, ovarian, brain, and hematopoietic malignancies^6–10^. Interestingly, the antitumor activity of FTY720 is not related to interaction with S1P receptor (S1PR)^11^. Several S1PR-independent signaling pathways have been reported to contribute to FTY720-induced apoptosis, including those mediated by protein kinase C δ^9^, RhoA-GTPase^12^, phosphatidylinositol 3-kinase (PI3K)^13^, protein phosphatase 2A^14^, vacuolar H^+^-ATPase^15^, signal transducer and activator of transcription (STAT)3^16^, Mcl-1^17^ and mitogen-activated protein (MAP) kinase^18^.

Somewhat unexpectedly, a recent study reported the pro-survival/anti-apoptotic effect of FTY720 in CCL39 lung fibroblasts and human umbilical vein endothelial cells through the upregulation of the anti-apoptotic protein Mcl-1, which is counter to the pro-apoptotic effect of FTY720 in chronic lymphotic leukemia cells^17, 19^. This dichotomous effect of FTY720 on apoptosis underlies cell type- and context-dependent differences in the signaling pathways governing apoptosis. Mcl-1 functions in tumorigenesis and radioresistance, particularly in solid tumors like OSCC, which suggests that Mcl-1 is a potential therapeutic target^20–22^. To investigate and validate the anti-tumor effect of FTY720 in OSCC in this study, we examined the efficacy and underlying mechanisms of FTY720 against oral cancer cells.

## Results

### FTY720 induces apoptosis through caspase-dependent and mitochondria pathways

**Figure 1:**
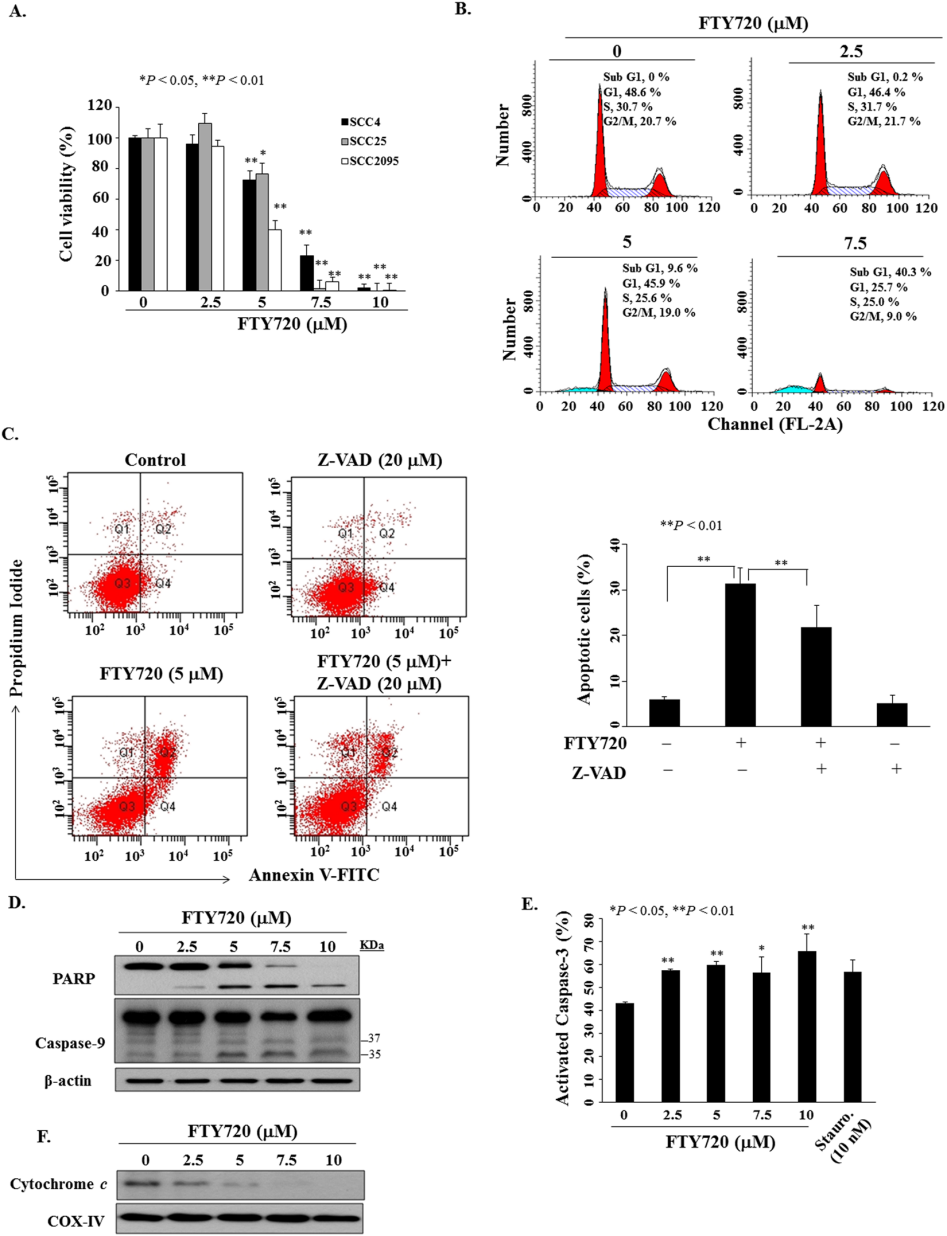
FTY720 induced cell death. (**A**) Effect of FTY720 at the indicated concentrations on the viability of oral cancer cells. Cells were treated with FTY720 in 5% FBS-supplemented DMEM/F12 medium in 96-well plates at 24 h, and cell viability was assessed by MTT assays. *Points*, means; *bars*, S.D. (n = 6). *P < 0.05, **P < 0.01 compared to the control group. (**B**) Cell cycle analysis showed an increased sub-G1 phase population in SCC2095 cells treated with FTY720 for 24 h. (**C**) Left, flow cytometric analysis of apoptotic death in SCC2095 cells after the treatment of FTY720 (5 μM) for 24 h with or without pretreatment of Z-VAD(OMe)-FMK (Z-VAD, 20 μM) for 1 h. Right, percentages in the graphs represent the percent of cells in the respective quadrants. *Columns*, means; *bar*, S.D. (n = 3). **P < 0.01. (**D**) Dose-dependent effect of FTY720 on caspase-9 activation and PARP cleavage in SCC2095 cells after 24 h exposure in 5% FBS-supplemented DMEM/F12 medium. (**E**) FTY720 induced a dose-dependent increase of activated caspase-3. *P < 0.05, **P < 0.01 when compared with the control group. Cells exposed to staurosporine (Stauro.) at 10 nM were used as a positive control. (**F**) Western blotting of mitochondrial extract.

Three oral cancer cell lines, SCC4, SCC25, and SCC2095, were used to investigate the anti-viability effect of FTY720. As shown in Fig. 1A, FTY720 treatment for 24 h decreased cell viability in the MTT assay in a dose-dependent manner. The IC_50_ values of FTY720 for SCC4, SCC25, and SCC2095 cells were 6.1, 6.3, and 4.5 µM, respectively. As the cell line most susceptible to FTY720, SCC2095 was used to assess the drug’s mode of antitumor action. Several lines of evidence indicated that the anti-viability activity of FTY720 on OSCC was, at least in part, attributable to its ability to induce apoptosis. First, propidium iodide (PI) staining revealed a dose-dependent accumulation of cells in the sub-G1 phase in response to FTY720 (Fig. 1B). Second, PI/Annexin V analysis demonstrated that FTY720-induced apoptosis could be partially rescued by pre-treatment with the caspase inhibitor Z-VAD(OMe)-FMK (Fig. 1C). Additional supporting evidence of apoptosis induction by FTY720 included PARP cleavage and caspase-9 activation (Fig. 1D), caspase-3 activation (Fig. 1E; staurosporine as a positive control), and cytochrome *c* release from mitochondria (Fig. 1F). Together, these data reveal that FTY720 induced caspase- and mitochondria-dependent apoptosis in SCC2095 cells.

### FTY720 downregulates Akt/NF-κB signaling and Mcl-1

**Figure 2:**
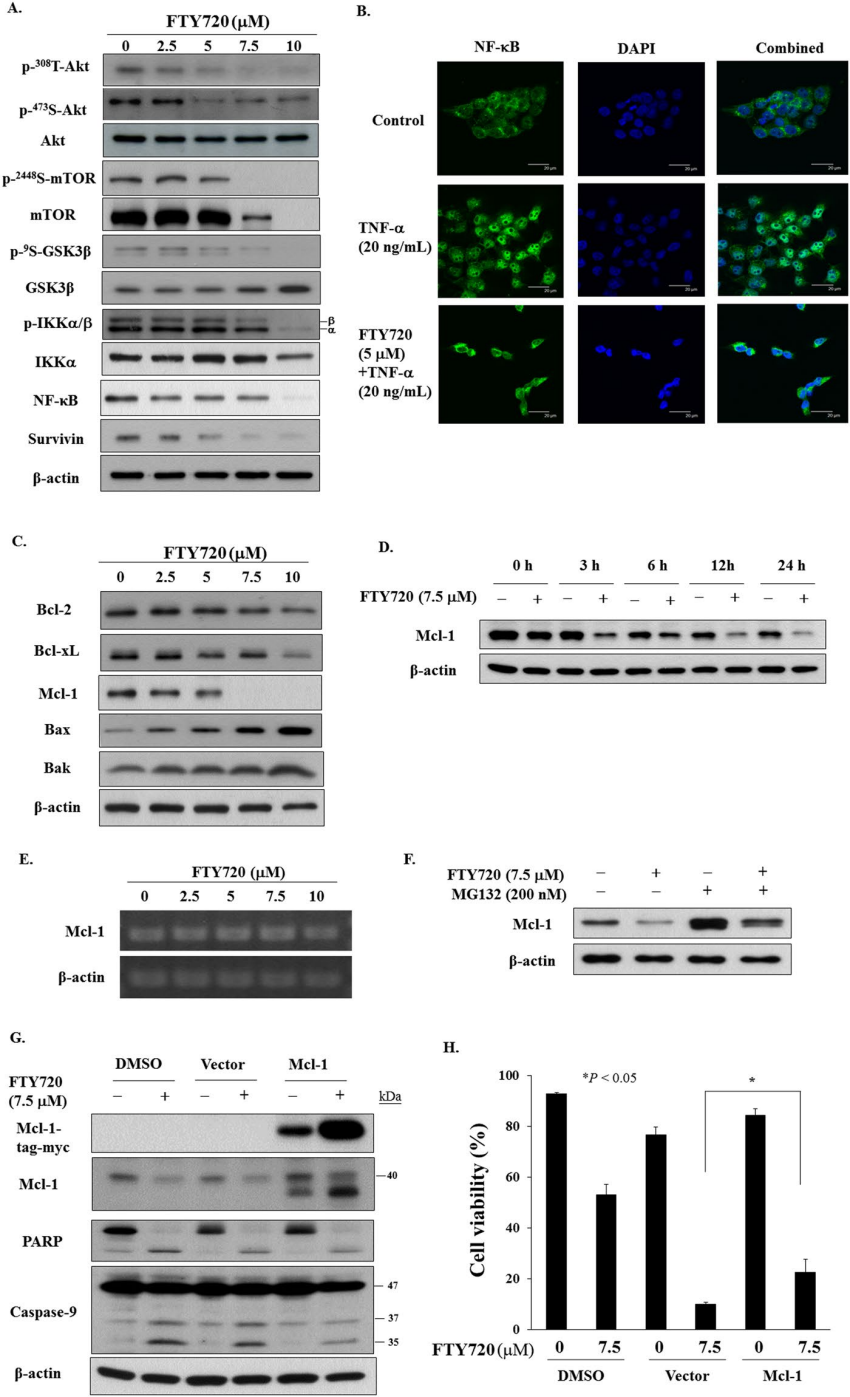
(**A**) Dose-dependent effects of FTY720 on the phosphorylation of Akt, mTOR, GSK3β, and IKKα/β, and the expression of NF-κB, and survivin in SCC2095 cells. Cells were treated with FTY720 in 5% FBS-supplemented DMEM/F12 medium for 24 h, and cell lysates were immunoblotted as described in Material and Methods. (**B**) Effect of FTY720 on the nuclear translocation of NF-κB. SCC2095 cells were treated with 5 μM FTY720 for 24 h, stained with anti-NF-κB, and examined by confocal microscopy. (**C**) Dose-dependent effects of FTY720 on the expression of Bcl-2, Bcl-xL, Mcl-1, Bax, and Bak. (**D**) Time-dependent effects of FTY720 on the expression of Mcl-1. (**E**) Mcl-1 mRNA expression measured by RT-PCR in cells treated with FTY720. (**F**) Effect of proteasome inhibitor on Mcl-1 expression. Cells were exposed to FTY720 in the presence or absence of 200 nM MG132 for 24 h. (**G**) Effect of ectopic Mcl-1 expression on apoptotic related proteins. SCC2095 cells were transfected with control vector or Mcl-1 plasmid for 24 h and then exposed to FTY720 for 24 h. Whole cell extracts were subjected to Western blot analysis. (**H**) Effect of Mcl-1 overexpression on the viability of SCC2095 treated with FTY720 (7.5 μM) for 24 h. After incubation, cells were analyzed using flow cytometry. *Columns*, means; *bar*, S.D. *P < 0.05.

The Akt/NF-κB signaling pathway plays a crucial role in regulating the invasive phenotype of oral cancer^23^. In light of the reported activity of FTY720 on inhibiting PI3K-mediated Akt phosphorylation in human hepatocellular carcinoma^13^, we assessed the effect of FTY720 on markers associated with this signaling axis. As shown in Fig. 2A, FTY720 suppressed the phosphorylation of Akt and its downstream effectors, glycogen synthase kinase (GSK) 3β, mammalian target of rapamycin (mTOR), and IκB kinase (IKK) in a dose-dependent manner in SCC2095 cells (Fig. 2A). The suppressive effect of FTY720 on NF-κB signaling was corroborated by two lines of evidence. First, the downregulation of the above signaling markers was accompanied by reduced expression of NF-κB and its downstream target survivin (Fig. 2A). Second, FTY720 (5 µM) effectively blocked tumor necrosis factor (TNF)-α-induced nuclear translocation of NF-κB (Fig. 2B).

Consistent with its ability to induce caspase-dependent apoptosis, FTY20 decreased the expression of Bcl-2, Bcl-xL and, to a greater extent, Mcl-1 in a dose- and/or time-dependent manner, accompanied by parallel increases in the proapoptotic proteins Bax and Bak (Fig. 2C and D). To further investigate the mechanism underlying the Mcl-1 downregulation, we measured Mcl-1 mRNA using quantitative RT-PCR and the proteasome inhibitor MG132. As shown, Mcl-1 mRNA levels remained unchanged in FTY720-treated cells (Fig. 2E). In contrast, the downregulation of Mcl-1 was abrogated by co-treatment with MG132 (Fig. 2F). The above findings suggest the involvement of proteasomal degradation in the Mcl-1 protein downregulation induced by FTY720 treatment.

Furthermore, we examined the effect of ectopic expression of Mcl-1 on FTY720-induced apoptotic death in SCC2095 cells. As shown in Fig. 2G and H, overexpression of Mcl-1 partially protected cells from FTY720-induced caspase-9 activation and PARP cleavage and cytotoxicity, suggesting that FTY720-induced apoptosis might, in part, be associated with its ability to suppress Mcl-1 expression. In addition, the co-treatment of Mcl-1 inhibitor A-1210477 further inhibited cell growth in FTY720-treated SCC2095 cells (Fig. S1).

### FTY720 induces autophagy

**Figure 3:**
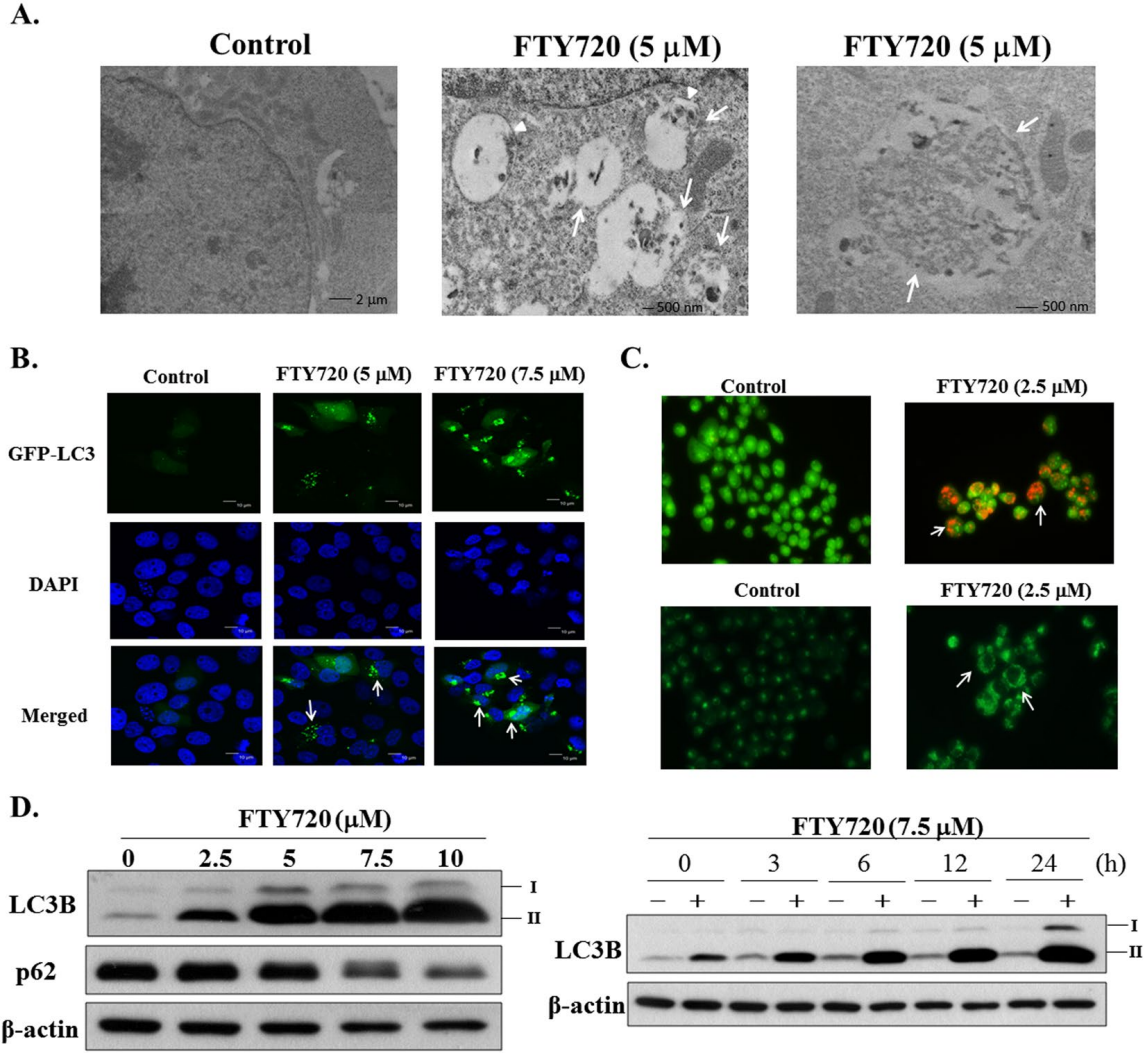
FTY720 induced autophagy. (**A**) Electron microscopic analysis of autophagosome formation in DMSO- or drug-treated SCC2095 cells as described in Materials and Methods. Middle panel: early autophagosomes (arrow). Note the double membrane of early autophagosome (arrowhead). Right panel: late autophagosomes (arrow). (**B**) SCC2095 cells expressing GFP-LC3 were treated with FTY720 at the indicated concentrations for 1 h and then fixed by 2.0% paraformaldehyde for confocal analysis. (**C**) Top, acridine orange staining. SCC2095 cells were treated with or without 2.5 μM FTY720 for 24 h and then stained with acridine orange (1 μg/mL), and then observed under a fluorescence microscope. Arrow: autophagosomes. Bottom, MDC staining. SCC2095 cells were treated with or without 2.5 μM FTY720 for 24 h, incubated with 0.05 mM MDC for 10 min and examined under a fluorescence microscope. (**D**) The expression of LC3B-II in SCC2095 cells. Left, the expression of LC3B-II and p62 after FTY720 treatment for 24 h in SCC2095 cells. Right, FTY720 at 7.5 μM induced LC3B-II accumulation in SCC2095 cells in a time-dependent manner.

In light of several reports that FTY720 induces autophagy in glioblastoma cells and ovarian cancer cells^10, 24^, we further investigated the potential interplay between autophagy and FTY720-mediated antitumor effects. Transmission electron microscopy revealed the ability of FTY720 to induce the formation of autophagosomes (arrow) in the cytoplasm, indicative of autophagy (Fig. 3A). This finding was confirmed by the fluorescent visualization of LC3 puncta formation^25^ in GFP-LC3-transfected SCC2095 cells under a confocal microscope (Fig. 3B). In addition, staining of cells with acridine orange and monodansylcadaverine (MDC) demonstrated the ability of FTY720 to induce the accumulation of acid vesticular organelles and autophagic vacuoles, respectively, in SCC2095 cells (Fig. 3C). Furthermore, Western blot analysis indicated dose- and time-dependent increases in the autophagosomal membrane-bound LC3B-II expression in FTY720-treated cells, and parallel decreases in the abundance of p62, a common component of protein aggregates that accumulate when autophagy is inhibited^26^ (Fig. 3D). Together, these results demonstrated the induction of autophagy.

### Inhibition of autophagy attenuates FTY720-induced cytotoxicity

**Figure 4:**
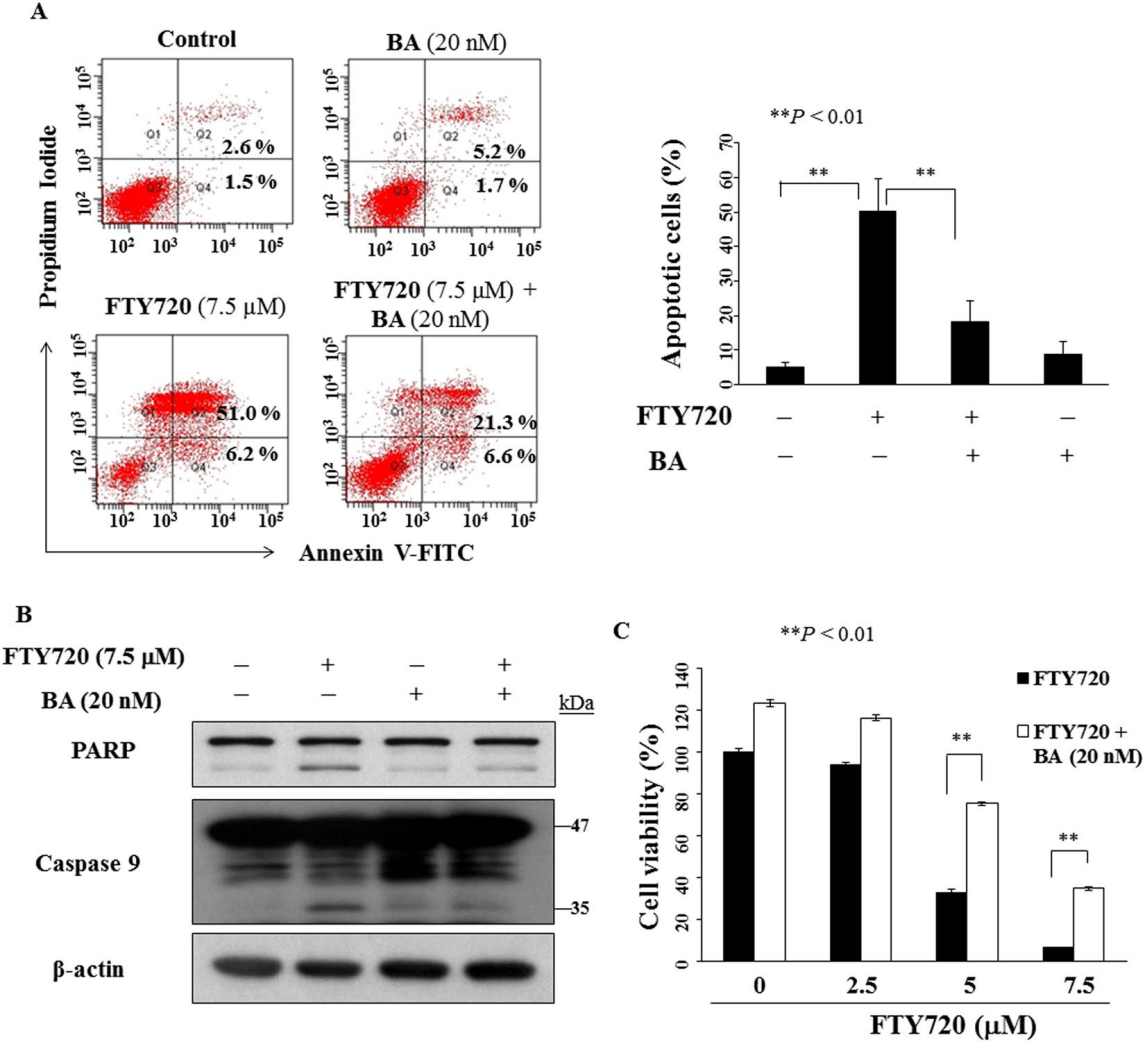
Effects of the autophagic inhibitor on FTY720-induced apoptosis. (**A**) Left, SCC2095 cells were treated with 7.5 μM FTY720 alone or in combination with 20 nM bafilomycin A1 (BA) for 24 h, and then Annexin V-FITC/PI double-staining analysis was performed. Right, percentages in the graphs represent the percent of cells in the respective quadrants. *Columns*, means; *bar*, S.D. **P < 0.01. (**B**) Effects of 7.5 μM FTY720 or 20 nM BA or the drug combination relative to DMSO control in SCC2095 cells after 24 h. (**C**) FTY720-inhibited cell growth could be blocked by BA. Cells were treated with FTY720 at indicated concentrations in the presence of 20 nM BA or DMSO for 24 h, and cell viability was determined by MTT assays. *Points*, means; *bar*, S.D. (n = 6). **P < 0.01.

Autophagy plays a protective or suppressive role in drug-induced cell death in a cell type- and/or context-specific manner^10, 27, 28^. To determine whether the autophagy induction played a pro-survival or pro-death role in OSCC cells, cells were co-treated with FTY720 and autophagy inhibitor bafilomycin A1 (BA; a vacuolar-type H^+^-ATPase inhibitor that blocks autophagosome-lysosome fusion) for 24 h, followed by annexin V-FITC/PI double-staining. As shown, FTY720-induced apoptosis was partially rescued by BA (Fig. 4A). Furthermore, cleaved PARP and caspase-9 activation could be partially rescued by the addition of BA in FTY720-treated cells (Fig. 4B). Equally important, co-treatment with BA protected cells from the suppressive effect of FTY720 on cell viability assessed in MTT assay (Fig. 4C). The results suggested that FTY720 induces autophagic cell death instead of protective autophagy.

### FTY720-mediated cytotoxicity is dependent on the generation of reactive oxygen species (ROS)

**Figure 5:**
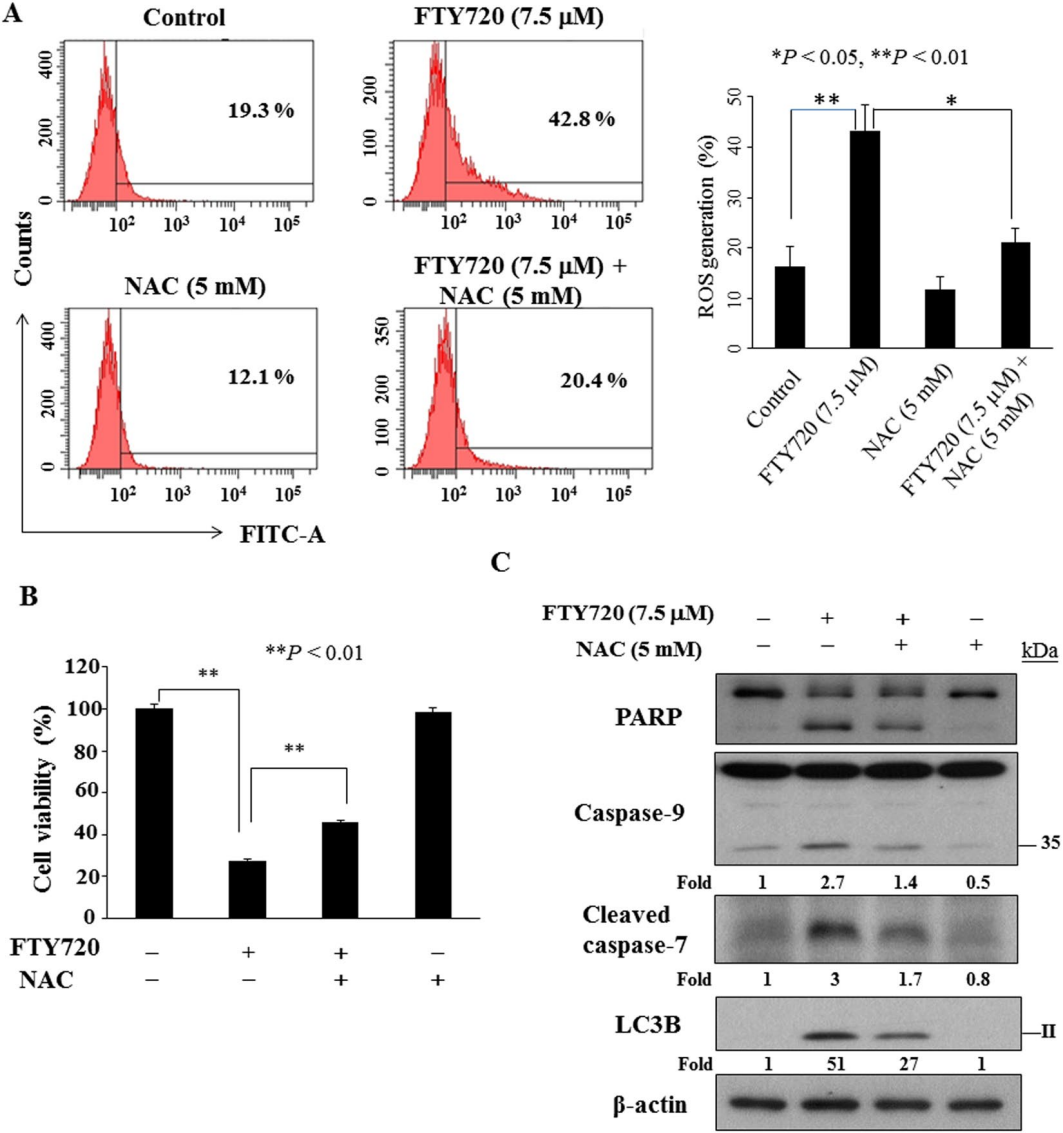
Reactive oxygen species analysis in SCC2095 cells. (**A**) Cells were treated with 7.5 μM FTY720 or DMSO for 6 h were stained with carboxy-DCFDA. *N*-acetylcysteine (NAC) was used to rescue ROS production. Data are presented as the mean ± S.D. (*n* = 4). (**B**) FTY720-inhibited cell growth could be blocked by NAC. Cells were treated with FTY720 (7.5 μM) in the presence of 5 mM NAC or DMSO for 6 h, and cell viability was determined by MTT assays. *Points*, mean; *bars*, S.D. (n = 6). **P < 0.01. (**C**) Effect of ROS inhibitor on PARP, caspase-9, cleaved caspase-7 and LC3B-II expression. Cells were exposed to FTY720 in the presence or absence of 5 mM NAC for 6 h. The values in fold or percentage denote the relative intensity of protein bands of drug-treated samples to that of the respective DMSO vehicle-treated control after being normalized to the internal reference (β-actin). Each value represents the average of two independent experiments.

As ROS generation represents a major mechanism by which many chemotherapeutic agents mediate their antitumor activities^29–31^, we investigated the effect of FTY720 on ROS accumulation in SCC2095 cells. Flow cytometric analysis using an H_2_DCFDA-based assay showed that treatment with FTY720 (7.5 µM) for 3 h led to a two fold increase in intracellular ROS, which could be abolished by the ROS scavenger *N*-acetylcysteine (NAC) (Fig. 5A). NAC treatment conferred partially protection against FTY720-induced cell death (Fig. 5B). Furthermore, NAC counteracted the effect of FTY720 on many apoptosis markers, including PARP cleavage, caspase-7, and caspase-9 activation. In addition, co-treatment with NAC partially rescued the expression of LC3B-II in FTY720-induced autophagy (Fig. 5C).

## Discussion

Although the *in vitro* and *in vivo* antitumor efficacy of FTY720 has been demonstrated in many types of cancer cells, the mechanism underlying its antitumor activity varies according to cell line and context. Clarification of this context is important in diagnosis and treatment decisions. In this study, we delineate the mechanism by which FTY720 mediates the tumor-suppressive effect on OSCC cells. In addition, FTY720 exhibits the unique ability to induce autophagy in OSCC cells, as characterized by LC3B-II conversion, reduced p62 expression, and autophagosome accumulation, through ROS production. Inhibition of autophagy by bafilomycin A1 protected OSCC cells from FTY720-induced apoptosis, suggesting a subtle interplay between two types of programmed cell death in mediating the antitumor effect of FTY720.

Our data indicate that FTY720 induces both caspase-dependent apoptosis and autophagic cell death in OSCC cells. Mechanistically, this effect was associated with ROS generation and proteasomal degradation of the antiapoptotic protein Mcl-1. It is documented that down-regulation of Mcl-1 sensitizes OSCC cells to radiation and chemotherapy^20, 32^. Because ectopic expression of Mcl-1 partially protected cells from FTY720-induced apoptosis, we rationalize that this Mcl-1 downregulation acts in concert with the inhibition of Akt/NF-kB signaling to facilitate caspase-dependent apoptosis in FTY720-treated OSCC cells.

Accumulating evidence demonstrates the unique redox situation in tumor cells and suggests the use of ROS generation as a strategy of anticancer therapy^33, 34^. Our data showed that FTY720 exhibits antitumor effects by increasing ROS generation in SCC2095 cells, and this inhibitory effect could be partially rescued by NAC.

In the literature, FTY720 has been reported to exhibit a dichotomous effect on autophagy. For example, FTY720 was shown to induce autophagy via a ROS-dependent mechanism, which promoted apoptosis in multiple myeloma cells^27^. This autophagy-related apoptosis was also reported to increase in FTY720-treated glioblastoma cells^24^. However, FTY720-mediated autophagy does not always lead to apoptosis, as autophagic flux in ovarian cancer cells induced by FTY-720 plays a cyto-protective role^10^. In a third cancer type, FTY720 was reported to block autophagy in mantle cell lymphoma, which enhanced the anticancer efficacy of milatuzumab^35^. These findings, together with our present study, highlight a complex, cell type-dependent mechanism by which FTY720 affects cancer cells. We have investigated the therapeutic benefits of treating oral squamous cell carcinoma with FTY720.

In conclusion, our data show that FTY720 induced downregulation of Akt-NF-κB pathway, ROS generation, Mcl-1 degradation, and autophagy-dependent apoptosis in OSCC cells. Collectively, these results demonstrate that the translational potential of FTY720 to foster novel therapeutic strategies for the treatment and prevention of human OSCC.

## Materials and Methods

### Reagents, Antibodies, and Plasmids

FTY720 (2-amino-2-[2-(4-octylphenyl)ethyl]propane-1,3-diol hydrochloride) was synthesized as described previously^36^. All agents were dissolved in DMSO, diluted in culture medium, and added to cells at a final DMSO concentration of 0.1%. Antibodies for the following biomarkers were obtained from Cell Signaling Technologies (Danvers, MA): Akt, p-^473^Ser Akt, p-^308^Thr Akt, cytochrome *c*, NF-κB, Mcl-1, Bcl-2, Bak, Bcl-xL, Bax, p-^9^Ser GSK3β, GSK3β, p-^2448^Ser mTOR, mTOR, LC3B, IKKα/β, survivin, p62, p-^176/180^Ser IKKα/β, cleaved caspase-7, PARP, and caspase-9. COX-IV antibody was purchased from Santa Cruz Biotechnology (Santa Cruz, CA); β-actin antibody, Sigma-Aldrich (St. Louis, MO). The GFP-LC3 plasmid was purchased from Addgene (Cambridge, MA). Mcl-1 plasmid was obtained from OriGene Technologies, Inc. (Rockville, MD). The enhanced chemiluminescence system for detection of immunoblotted proteins was from GE Healthcare (Little Chalfont, Buckinghamshire, UK). Other chemicals and reagents were obtained from Sigma-Aldrich unless otherwise noted.

### Cell Culture

OSCC cell lines SCC4, SCC25, and SCC2095 were kindly provided by Professor Susan R. Mallery (The Ohio State University). All cells were cultured in DMEM/F12 (Invitrogen, Carlsbad, CA) and supplemented with 10% heat-inactivated fetal bovine serum (FBS; Gibco, Grand Island, NY) and penicillin (100 U/ml)/streptomycin (100 μg/ml) (Invitrogen). All cell types were cultured at 37 °C in an atmosphere of 5% CO_2_.

### Cell Viability Analysis

Cell viability was assessed using the 3-(4,5-dimethylthiazol-2-yl)-2,5-diphenyltetrazolium bromide (MTT) assay in 6 replicates as described previously^37^. In brief, cells were seeded at a density of 5 × 10^3^ cells per well in 96-well flat-bottomed plates; 24 h later, cells were treated with FTY720 or DMSO vehicle at the concentrations indicated in the individual figures. After 24 h, the medium was removed, replaced by 200 µL DMEM/F12 containing 0.5 mg/mL of MTT, and cells were incubated in the CO_2_ incubator at 37 °C for 2 h. Supernatants were aspirated from the wells, and the reduced MTT dye was solubilized in 200 µL/well DMSO. Absorbance at 570 nm was measured using a plate reader.

### Immunoblotting

Western blot analysis was performed as reported previously^38^. Briefly, treated cells were washed with phosphate-buffered saline (PBS), resuspended in SDS sample buffer, sonicated for 5 sec, and then boiled for 5 min. After brief centrifugation, equivalent amounts of proteins from the soluble fractions of cell lysates were resolved in 10% SDS-polyacrylamide gels on a Minigel apparatus, and transferred to a nitrocellulose membrane using a semidry transfer cell. The transblotted membranes were washed thrice with TBS containing 0.05% Tween 20 (TBST). After blocking with TBST containing 5% nonfat milk for 120 min, the membranes were incubated with the appropriate primary antibodies at 1:500 dilution (with the exception of anti–β-actin antibody, 1:2,000) in TBST–5% low fat milk at 4 °C overnight, and then washed thrice with TBST. Membranes were probed with goat anti-rabbit or anti-mouse IgG-horseradish peroxidase conjugates (1:2,500) for 90 min at room temperature, and washed thrice with TBST. The immunoblots were visualized by enhanced chemiluminescence.

### Flow Cytometry

Cells (2 × 10^5^/3 mL) were treated with the indicated concentration of FTY720 or DMSO for 24 hours. After being washed twice with ice-cold PBS, cells were fixed in 70% cold ethanol for 4 hours at 4 °C. For cell cycle analysis, cells were stained with propidium iodide (PI) and analyzed by multicycler software. Caspase-3 activation was assessed using a FITC rabbit anti-active caspase-3 kit (BD Pharmingen) according to the manufacturer’s protocol. ROS production was detected using the fluorescence probe 5-(and-6)-carboxy-2′, 7′-dichlorodihydrofluoresceindiacetate (carboxy-DCFDA)^39^. For apoptosis evaluation, cells were stained with Annexin V and PI (1 μg/mL), counted on a BD FACSAria flow cytometer, and analyzed by ModFitLT V3.0 software program (Becton Dickinson, Germany).

### Cytochrome c release from Mitochondria

Cells (2 × 10^5^/3 mL) were treated with FTY720 at the indicated concentrations for 24 h. The mitochondria isolation kit (Pierce, Rockford, IL) was used according to the manufacturer’s instructions to obtain the mitochondrial fraction. Cytochrome *c* was detected by Western blot analysis using COX IV as the mitochondrial loading control.

### Transient Transfection for Confocal Imaging and Overexpression

Cells (2 × 10^5^/3 mL) were plated on cover slips in a six-well plate. For the nuclear translocation of NF-κB^40^, the cells were treated with 5 μM FTY720 for 24 h with or without 20 nM tumor necrosis factor-α (TNF-α) for 30 min. The cells were fixed in 2% paraformaldehyde for 30 min at room temperature and permeabilized with 0.1% Triton X-100 for 20 min. After blocking with 1% bovine serum albumin (BSA), the cells were incubated with human NF-κB antibody overnight at 4 °C, followed by incubation with anti-rabbit IgG for 1 h at room temperature. Cells were washed with TBST and then covered before undergoing fluorescent microscopic examination. For the confocal examination of GFP-LC3^41^, cells were transfected with 1 μg GFP-LC3 plasmid, followed by the indicated concentrations of FTY720. Cells were fixed in 2% paraformaldehyde for 30 min at room temperature, and permeabilized with 0.1% Triton X-100 for 20 min. Cells were washed with PBS and then covered before undergoing fluorescent microscopic examination. For overexpression of Mcl-1, SCC2095 cells (2 × 10^5^/3 mL) were transfected with Fugene HP (Roche) according to the manufacture’s protocol and then cultured in a six-well plate for 24 h. Plasmids expressing vector (pCMV6-Entry) and Mcl-1 (Myc-DDK-tagged) were purchased from OriGene Technology (Rockville, MD). Proteins were collected for Western blot analysis.

### Detection of Autophagosome Formation with Acridine Orange and Monodansylcadaverine (MDC)

To detect the presence of acidic vesicular organelles, drug-treated cells were stained with acridine orange (1 μg/mL) for 15 min and examined under a fluorescence microscope. For MDC staining, drug-treated cells were incubated with 50 μM MDC for 10 min at 37 °C and the observed under a fluorescence microscope.

### RNA isolation and Semiquantitative PCR

After vehicle or drug treatments, SCC2095 cells were subjected to total RNA isolation using Trizol reagent (Invitrogen). Total RNA was then reverse transcribed to cDNA using the RevertAid First strand cDNA Synthesis kit (Fermentas, Thermo Scientific), respectively according to the manufacture’s instructions. RNA concentrations were determined by measured absorption at 260 nm in a spectrophotometer. The sequences of the PCR primers used were as follows: Mcl-1, 5′-CTTACGACGGGTTGGG-3′/5′-GGTTCGATGCAGCTTTCTTGG-3′ and GADPH, 5′-CAGCCTCAAGATCATCAGCA-3′/5′-ACAGTCTTCTGGGTGGCAGT-3′. PCR products were separated electrophoretically in 2% agarose gels and visualized by ethidium bromide staining.

### Transmission Electron Microscope

Samples were performed as reported previously^42^. Briefly, cells were fixed in a solution containing 0.2 M sodium cacodylate, 2% paraformaldehyde, 2.5% glutaraldehyde for 1 h. After fixing, cells were suspended in a buffered solution containing 1% osmic acid for 1 h, followed by dehydration in a graded ethanol series, washing with acetone and embedding into EPON epoxy resin. Ultrathin sections (60–80 nm) were prepared on an ultramicrotome and double-stained with lead citrate and uranyl acetate. All sections were examined and photographed with a Hitachi H-600 transmission electron microscope (Hitachi, Tokyo, Japan).

### Statistical Analysis

All experiments were performed in three replicates. Statistical significance was determined with Student’s *t* test comparison between two groups of data sets. Differences between groups were considered significant at *P < 0.05, **P < 0.01.

## Electronic supplementary material

Supplementary information 
