# Author Correction: FTY720 Induces Autophagy-Associated Apoptosis in Human Oral Squamous Carcinoma Cells, in Part, through a Reactive Oxygen Species/Mcl-1-Dependent Mechanism

**DOI:** 10.1038/s41598-020-60297-8

**Published:** 2020-02-19

**Authors:** Li-Yuan Bai, Chang-Fang Chiu, Shih-Jiuan Chiu, Po-Chen Chu, Jing-Ru Weng

**Affiliations:** 1 College of Medicine, China Medical University, Taichung, 40402 Taiwan; 2 Division of Hematology and Oncology, Department of Internal Medicine, China Medical University Hospital, Taichung, 40447 Taiwan; 3 Cancer Center, China Medical University Hospital, Taichung, 40447 Taiwan; 4 School of Pharmacy, Taipei Medical University, Taipei, 11042 Taiwan; 5 Institute of Biological Chemistry, Academia Sinica, Taipei, 11529 Taiwan; 6 Department of Marine Biotechnology and Resources, National Sun-Yat-sen University, Kaohsiung, 80424 Taiwan

Correction to: *Scientific Reports* 10.1038/s41598-017-06047-9, published online 17 July 2017

In the original version of this Article, there was an error in Affiliation 6 which was incorrectly listed as ‘Department of Marine Technology and Resources, National Sun-Yat-sen University, Kaohisung, 80424, Taiwan’. The correct affiliation is listed below:

Department of Marine Biotechnology and Resources, National Sun-Yat-sen University, Kaohsiung, 80424, Taiwan

This error has now been corrected in the PDF and HTML versions of the Article, and in the accompanying Supplementary information file

