# Supplementary material for: FTY720 Induces Autophagy-Associated Apoptosis in Human Oral Squamous Carcinoma Cells, in Part, through a Reactive Oxygen Species/Mcl-1-Dependent Mechanism
Source: Sci Rep. 2017 Jul 17;7:5600. doi: 10.1038/s41598-017-06047-9 (PMC5514089; doi:10.1038/s41598-017-06047-9)
Supplement: Supplementary file 1 — Supplementary information [file 41598_2017_6047_MOESM1_ESM.pdf]

Supporting Information

FTY720 Induces Autophagy-Associated Apoptosis in Human Oral Squamous Carcinoma Cells, in Part, through a Reactive Oxygen Species/Mcl-1-Dependent Mechanism

Li-Yuan Bai[1,2], Chang-Fang Chiu[2,3], Shih-Jiuan Chiu[4], Po-Chen Chu[5], and Jing-Ru Weng[6,*]

[1]College of Medicine, China Medical University, Taichung 40402, Taiwan; [2]Division of Hematology and Oncology, Department of Internal Medicine, China Medical University Hospital, Taichung 40447, Taiwan; [3]Cancer Center, China Medical University Hospital, Taichung 40447, Taiwan; [4]School of Pharmacy, Taipei Medical University, Taipei 11042, Taiwan; [5]Institute of Biological Chemistry, Academia Sinica, Taipei 11529, Taiwan; [6]Department of Marine Biotechnology and Resources, National Sun Yat-sen University, Kaohsiung 80424, Taiwan

Figure		Page
S1	Effects of 5 μM FTY720, 100 nM A-1210477, or both drugs relative to DMSO control in SCC2095 cells after 24 h. Cells were treated with FTY720 at indicated concentrations in the presence of 100 nM A-1210477 or DMSO for 24 h, and cell viability was determined by MTT assay. *Points*, means; *bar*, S.D. (n = 6). $^*P < 0.05$.	4

Supplementary materials and methods

Effects of 5 μM FTY720, 100 nM A-1210477, or both drugs relative to DMSO control in SCC2095 cells after 24 h.

Cells ($5x10^3$) were treated with FTY720 (5 μM) in the presence of 100 nM A-1210477 or DMSO in 5% FBS-supplemented DMEM/F12 medium in 96-well plates. After 24 h, the medium was removed, replaced by 200 μL DMEM/F12 containing 0.5 mg/mL of MTT, and cells were incubated in the CO_2 incubator at 37°C for 2 h. Supernatants were aspirated from the wells, and the reduced MTT dye was solubilized in 200 μL/well DMSO. Absorbance at 570 nm was measured using a plate reader.

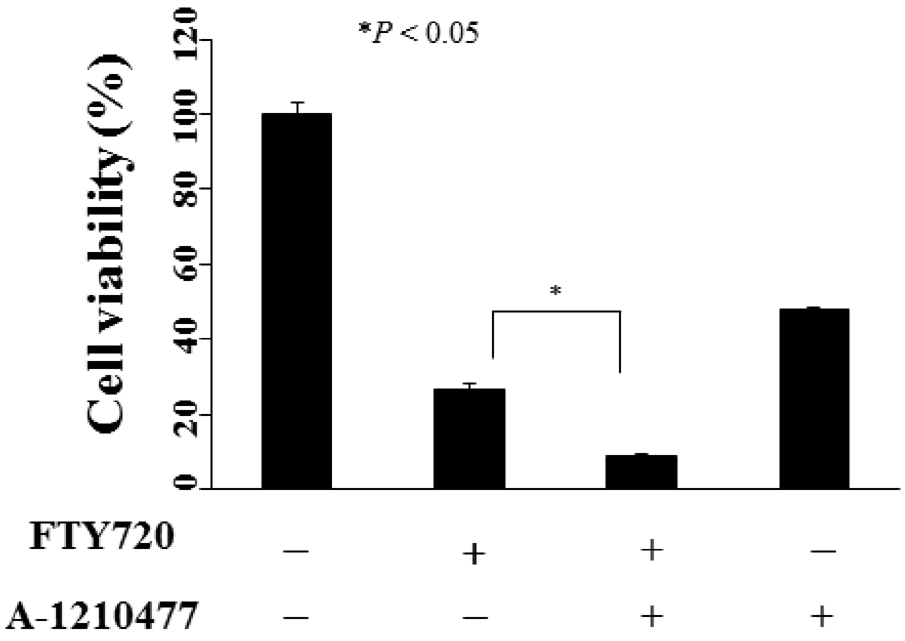

*P < 0.05
Cell viability (%)
0
20
40
60
80
100
120
*
FTY720
−
+
+
−
A-1210477
−
−
+
+
